# Niacin Reduces Atherosclerosis Development in APOE*3Leiden.CETP Mice Mainly by Reducing NonHDL-Cholesterol

**DOI:** 10.1371/journal.pone.0066467

**Published:** 2013-06-19

**Authors:** Susan Kühnast, Mieke C. Louwe, Mattijs M. Heemskerk, Elsbet J. Pieterman, Jan B. van Klinken, Sjoerd A. A. van den Berg, Johannes W. A. Smit, Louis M. Havekes, Patrick C. N. Rensen, José W. A. van der Hoorn, Hans M. G. Princen, J. Wouter Jukema

**Affiliations:** 1 TNO - Metabolic Health Research, Leiden, The Netherlands; 2 Department of Cardiology, Leiden University Medical Center, Leiden, The Netherlands; 3 Department of Endocrinology and Metabolic Diseases, Leiden University Medical Center, Leiden, The Netherlands; 4 Department of Human Genetics, Leiden University Medical Center, Leiden, The Netherlands; 5 Einthoven Laboratory for Experimental Vascular Medicine, Leiden University Medical Center, Leiden, The Netherlands; King’s College London School of Medicine, United Kingdom

## Abstract

**Objective:**

Niacin potently lowers triglycerides, mildly decreases LDL-cholesterol, and largely increases HDL-cholesterol. Despite evidence for an atheroprotective effect of niacin from previous small clinical studies, the large outcome trials, AIM-HIGH and HPS2-THRIVE did not reveal additional beneficial effects of niacin (alone or in combination with laropiprant) on top of statin treatment. We aimed to address this apparent discrepancy by investigating the effects of niacin without and with simvastatin on atherosclerosis development and determine the underlying mechanisms, in APOE*3Leiden.CETP mice, a model for familial dysbetalipoproteinemia (FD).

**Approach and Results:**

Mice were fed a western-type diet containing cholesterol without or with niacin (120 mg/kg/day), simvastatin (36 mg/kg/day) or their combination for 18 weeks. Similarly as in FD patients, niacin reduced total cholesterol by -39% and triglycerides by −50%, (both P<0.001). Simvastatin and the combination reduced total cholesterol (−30%; −55%, P<0.001) where the combination revealed a greater reduction compared to simvastatin (−36%, P<0.001). Niacin decreased total cholesterol and triglycerides primarily by increasing VLDL clearance. Niacin increased HDL-cholesterol (+28%, P<0.01) and mildly increased reverse cholesterol transport. All treatments reduced monocyte adhesion to the endothelium (−46%; −47%, P<0.01; −53%, P<0.001), atherosclerotic lesion area (−78%; −49%, P<0.01; −87%, P<0.001) and severity. Compared to simvastatin, the combination increased plaque stability index [(SMC+collagen)/macrophages] (3-fold, P<0.01). Niacin and the combination reduced T cells in the aortic root (−71%, P<0.01; −81%, P<0.001). Lesion area was strongly predicted by nonHDL-cholesterol (R^2^ = 0.69, P<0.001) and to a much lesser extent by HDL-cholesterol (R^2^ = 0.20, P<0.001).

**Conclusion:**

Niacin decreases atherosclerosis development mainly by reducing nonHDL-cholesterol with modest HDL-cholesterol-raising and additional anti-inflammatory effects. The additive effect of niacin on top of simvastatin is mostly dependent on its nonHDL-cholesterol-lowering capacities. These data suggest that clinical beneficial effects of niacin are largely dependent on its ability to lower LDL-cholesterol on top of concomitant lipid-lowering therapy.

## Introduction

The beneficial effects of niacin, also known as nicotinic acid or vitamin B3, on plasma lipids and lipoproteins were first described in the 1950s [1]. According to a meta-analysis of 30 randomized controlled trials, niacin potently reduced triglycerides (TG) by ∼15–30% and increased HDL-cholesterol (HDL-C) by ∼10–25%, while mildly reducing plasma total cholesterol (TC) by ∼5–15% and LDL-cholesterol (LDL-C) by ∼5–20%, suggesting an atheroprotective effect [2]. Whereas previous small clinical studies supported this notion [3]–[6], the recent large outcome trials AIM-HIGH and HPS2-THRIVE failed to reveal additional beneficial effects of niacin on top of statin treatment [7], [8].

In patients with atherosclerotic disease or those at risk for atherosclerotic disease due to dyslipidemia, the primary goal of lipid-modifying therapy is the lowering of LDL-C [9]. To this end, statins are currently the standard treatment for cardiovascular disease (CVD) resulting in a 25–45% risk reduction for cardiovascular events [10]. However, a substantial residual risk for adverse cardiovascular outcomes remains with statin therapy [3], [11]. Moreover, despite maximally tolerated statin treatment, some patients cannot reach LDL-C goals. This high risk population together with statin intolerant patients verify the need for another LDL-C-lowering agent to (further) reduce LDL-C levels [12]. Treatment of low HDL-C is currently considered a secondary lipid target in the reduction of cardiovascular risk [3], since low HDL-C is an independent risk factor for CVD [2], [13]. Considering the current treatment options, the question remains whether to further reduce LDL-C or to increase HDL-C in addition to LDL-C-lowering [12]. Therefore, due to both its nonHDL-C-lowering and HDL-C-raising properties, niacin was considered an attractive candidate for further cardiovascular risk reduction in addition to statin therapy.

Indeed, an initial small clinical study suggested that the addition of niacin to statin treatment may cause potentially clinical significant reductions in relative risk of cardiovascular events [6]. Recently a number of relatively small secondary prevention studies (ARBITER-2 [4], ARBITER-3 [5] and ARBITER-6-HALTS [3]) have shown reduced progression and even regression of atherosclerosis with combination treatment of niacin and statins compared to statins alone, as measured by carotid artery intima media thickness as a surrogate for clinical endpoints. Magnetic resonance imaging results from another study confirmed the reduction in carotid atherosclerosis with niacin in statin-treated patients [14]. These clinical data were corroborated by recent observations that niacin reduces atherosclerosis development, independent of lipid-lowering or HDL-C-elevation, in hyperlipidemic LDL receptor knockout mice on a high fat diet containing 1.5% cholesterol [15]. Despite these promising data, the large outcome trial, AIM-HIGH, addressing the effect of niacin on top of aggressive LDL-lowering treatment, has recently been prematurely terminated due to futility [8]. In accordance, the much larger HPS2-THRIVE trial failed to reveal additional risk reduction of cardiovascular events with extended-release (ER)-niacin/laropiprant in combination with statin treatment as compared to statin mono-treatment [7]. ER-niacin [2] and ER-niacin/laropiprant combination treatment [16] are more tolerable formulations that have been developed due to a reluctance to use niacin for clinical treatment as a result of extreme flushing as a side effect [17].

In the present study, we aimed to address the seeming discrepancy between the beneficial effects of niacin in initial clinical trials, [3]–[6], [14] as well as in LDL receptor knockout mice, a model irresponsive to the lipid-modulating effects of niacin [15], and the lack of effect of niacin on top of statin treatment on reduction of cardiovascular events in the AIM-HIGH [8] and HPS2-THRIVE trials [7]. Therefore, we evaluated the effects of niacin without and with simvastatin on atherosclerosis development and investigated the underlying mechanisms and contributing factors in APOE*3Leiden.CETP mice. This is a well-established mouse model for familial dysbetalipoproteinemia (FD) with human-like lipoprotein metabolism and atherosclerosis development. These mice respond to the lipid-lowering effects of both niacin [18] and statins, e.g. atorvastatin [19], as well as the HDL-C-raising effect of niacin [18].

## Methods

### Animals, Diets and Experimental Design

Female APOE*3Leiden.CETP transgenic mice [20], expressing human cholesteryl ester transfer protein (CETP) under control of its natural flanking regions, were housed under standard conditions with a 12 h light-dark cycle and had free access to food and water during the experiment unless indicated otherwise. Body weight (BW) and food intake were monitored during the entire study. To increase plasma cholesterol levels up to ∼12 mmol/L, 8–12 week-old mice were fed a semi-synthetic cholesterol-rich diet, containing 15% (w/w) cacao butter and 0.1% cholesterol (Western-type diet; Hope Farms, Woerden, The Netherlands) for 3 weeks. After matching based on age, BW, TC, TG and HDL-C mice (n = 15 per group) received a control western-type diet (WTD) without or with 0.1% (w/w) niacin (120 mg/kg/day), 0.03% (w/w) simvastatin (36 mg/kg/day) or their combination for 18 weeks. During the treatment period, the effects of treatment on plasma lipids, lipoprotein profiles, CETP activity and CETP mass were assessed at the indicated time points.

The dose of simvastatin targeted a 30–35% reduction in TC and that of niacin a 20–30% increase in HDL-C. While we achieved these targets, it should be noted that the dose of simvastatin was 3 times higher than the maximum dose used in the clinic taking into account a 10 times faster metabolism in mice. For niacin the dose was comparable to that in patients, about 1 g/day. At the end of the experiment all animals were sacrificed by CO_2_ inhalation. Liver and white adipose tissue (WAT) were isolated to assess CETP expression (n = 6–8 per group) and hearts were isolated to assess atherosclerosis development (n = 15 per group). Separate additional experiments were performed to evaluate the effects of niacin on VLDL production and clearance, as well as reverse cholesterol transport (RCT). Animal experiments were approved by the Institutional Animal Care and Use Committee of The Netherlands Organization for Applied Research (TNO).

### Plasma Lipids and Lipoprotein Profiles

After a 4 h fast, blood was collected via tail vein bleeding and plasma was isolated. Plasma TC, HDL-C after precipitation of apoB-containing lipoproteins using MnCl_2_ [21] and TG were determined individually using enzymatic kits 1489437 and 1488872 (both from Roche diagnostics), according to the manufacturer’s protocols. After 4 and 18 weeks of treatment, pooled lipoprotein profiles for TC were measured by fast protein liquid chromatography (FPLC) [20].

### VLDL Production and Clearance Experiments

APOE*3Leiden.CETP mice (11–14 weeks of age) were fed a WTD containing 0.1% cholesterol for 3 weeks. Upon subsequent matching according to plasma TC and TG levels, mice received the cholesterol-containing WTD without or with 0.1% (w/w) niacin for an additional 3 weeks [18] and VLDL production and clearance were determined as described [22].

Plasma was obtained via tail vein bleeding in heparin microvettes for randomization (Sarstedt, Germany) and in chilled paraoxon-coated capillary tubes to prevent *ex vivo* TG hydrolysis for VLDL production and clearance, and assayed for TG and TC using commercially available kits 1488872 and 236691 (Roche Molecular Biochemicals, Indianapolis, IN, USA), respectively.

For the VLDL production experiment, 6 control and 6 niacin-treated mice were fasted for 4 h. During the experiment, mice were sedated with 6.25 mg/kg acepromazine (Alfasan), 6.25 mg/kg midazolam (Roche) and 0.31 mg/kg fentanyl (Janssen-Cilag). At t = 0 min, blood was taken via tail bleeding and mice were injected intravenously with 100 µL PBS containing 100 µCi Trans^35^S label to measure de novo total apoB synthesis. After 30 min, the mice received 500 mg of tyloxapol (Triton WR-1339, Sigma-Aldrich) per kg BW as a 10% (w/w) solution in sterile saline, to prevent systemic lipolysis of newly secreted hepatic VLDL-TG. Additional blood samples were taken 15, 30, 60, and 90 min after tyloxapol injection and used for determination of plasma TG concentration. After 120 min, the mice were sacrificed and blood was collected by orbital puncture for isolation of VLDL by density gradient ultracentrifugation. Incorporation of ^35^S-label was measured in the VLDL fraction as marker of *de novo* apoB synthesis.

For the VLDL clearance experiment, glycerol tri[^3^H]oleate (triolein, TO)- and [1α,2α(n)-^14^C]cholesteryl oleate (CO)-double labeled VLDL-like emulsion particles (80 nm) were used [23]. In short, radiolabeled emulsions were obtained by adding 100 µCi of [^3^H]TO and 10 µCi of [^14^C]CO to 100 mg of emulsion lipids before sonication (isotopes obtained from GE Healthcare, Little Chalfont, U.K.). APOE*3Leiden.CETP mice (5 control and 5 niacin-treated mice) were fasted for 4 h, sedated as described above, and injected intravenously with the radiolabeled emulsion particles (1.0 mg TG in 200 µL PBS). Blood was taken from the tail vein to determine the content of [^3^H]TO and [^14^C]CO in serum at 2, 5, 10 and 15 min after emulsion injection. Fifteen min after injection, plasma was collected by orbital puncture and mice were sacrificed by cervical dislocation. Organs were harvested and saponified to determine uptake of radioactivity derived from [^3^H]TO and [^14^C]CO by various organs [22].

### Endogenous CETP Activity, CETP Mass and CETP mRNA Expression Analysis

Plasma endogenous CETP activity was determined by a fluorescent method using donor liposomes enriched with nitrobenzoxadiazole-labeled cholesteryl esters (RB-CETP, Roar Biomedical, New York, NY) as described [19]. CETP activity was calculated as nmol cholesteryl ester transfer/mL plasma/h. Plasma CETP mass was determined by using the DAIICHI CETP ELISA kit according to manufacturer’s instructions (Daiichi, Tokyo, Japan) [18]. Total RNA was extracted from liver and white adipose tissue (WAT) using an RNA isolation kit according to manufacturer’s specifications (Macherey-Nagel, Düren, Germany). Total RNA concentrations were measured with Nanodrop. One µg of RNA was reversed-transcribed to cDNA with iScriptcDNA Synthesis kit (Bio-Rad) and purified with Nucleospin Extract II kit (Macherey-Nagel, Düren, Germany). Real-time PCR (RT-PCR) was carried out on an iQ5 PCR detection system (Bio-Rad) using Sensimix SYBR Green RT-PCR mix (Quantace, London, UK). Hypoxanthine-guanine phosporibosyltransferase (HPRT) and acidic ribosomal phosphoprotein PO (36B4) were used as the standard housekeeping genes and expression levels were normalized to these housekeeping genes. Primer sequences are listed in Table S1.

### Histological Assessment of Atherosclerosis

After isolation, hearts were fixed in formalin, embedded in paraffin and cross-sectioned (5 µm) throughout the aortic root area. For each mouse, four sections at intervals of 50 µm were used for quantitative and qualitative assessment of the atherosclerotic lesions after staining with hematoxylin-phloxin-saffron. For determination of severity of atherosclerosis, the lesions were classified into five categories: I) early fatty streak, II) regular fatty streak, III) mild plaque, IV) moderate plaque, and V) severe plaque according to the American Heart Association classification [19], [24]. Lesion severity as a percentage of the number of lesions was calculated. To this end, type I–III lesions were classified as mild lesions and type IV–V lesions were classified as severe lesions. Total lesion area and number of lesions per cross section, as well as the percentage undiseased segments, were calculated. In each segment used for lesion quantification, the number of monocytes adhering to the endothelium and the numbers of T cells in the total aortic root area were counted after immunostaining with AIA 31240 antibody (1∶1000; Accurate Chemical and Scientific, New York, New York, USA) and CD3 (1∶500; AbD Serotec, Oxford, UK), respectively. Macrophage content of the lesions was measured after immunostaining with Mac-3 (1∶50; BD Pharmingen, the Netherlands). In addition, sirius red staining was used to quantify the collagen content in the plaque [25] and the antibody alpha actin (1∶800; DAKO, Glostrup, Denmark) was used to quantify the smooth muscle cell (SMC) content [26]. Stained areas were measured using Cell D imaging software (Olympus Soft Imaging Solutions).

### Reverse Cholesterol Transport Experiment

16 recipient APOE*3Leiden.CETP mice (10–12 weeks of age) were fed a WTD containing 0.1% cholesterol for a run-in period of 3 weeks after which they were subdivided into 2 groups according to age, BW, TC, TG and HDL-C. After matching, mice (n = 8 per group) received a control cholesterol-containing WTD without or with 0.1% (w/w) niacin (120 mg/kg/day) for 3 weeks.

6 donor APOE*3Leiden.CETP mice (10–12 weeks of age) fed a WTD containing 0.1% cholesterol for 3 weeks were injected intraperitoneally with 1 mL solution of 3% thioglycollate to induce an inflammatory response. Three days after the injection, mice were injected intraperitoneally with approximately 300 µCi [^3^H]-cholesterol together with 100 µg/mL acetylated LDL. Mice were sacrificed 1 h later by CO_2_ inhalation. [^3^H]-cholesterol-labeled macrophages were collected from the 6 donor mice by peritoneal lavage. These macrophages were washed twice with cold PBS and injected intraperitoneally into the 16 recipient APOE*3Leiden.CETP mice. Each recipient mouse received 2.8×10^6^ [^3^H]-cholesterol-labeled macrophages containing 7.8×10^6 ^dpm [^3^H]-cholesterol. Mice were individually caged for 48 h in order to collect feces and sacrificed by CO_2_ inhalation. ^3^H-activity was determined in the plasma, liver and feces. The *in vivo* RCT experiment was based on methods previously described [27], [28].

### Statistical Analyses

Significance of differences between the groups was calculated non-parametrically using a Kruskal-Wallis test followed by a Mann-Whitney U-test for independent samples. We performed a univariate analysis of variance (ANOVA) to investigate the role of TC, nonHDL-C and HDL-C exposure as contributing factors in lesion development. A two-way analysis of covariance (ANCOVA) was performed to test for group differences in lesion area, monocyte adhesion, T cell abundance and macrophage area after correcting for HDL-C and nonHDL-C exposure. SPSS 17.0 for Windows (SPSS, Chicago, USA) was used for statistical analysis. All groups were compared to the control group and the combination group was also compared to the simvastatin group. Values are presented as means ± SD. P-values <0.05 were considered statistically significant. In the figures, the symbol * is used to compare to the control group, and # to compare to the simvastatin group.

## Results

### Niacin, Simvastatin and their Combination Reduce Plasma Total Cholesterol and Triglycerides and Niacin Increases HDL-C in APOE*3Leiden.CETP Mice

**Figure 1:**
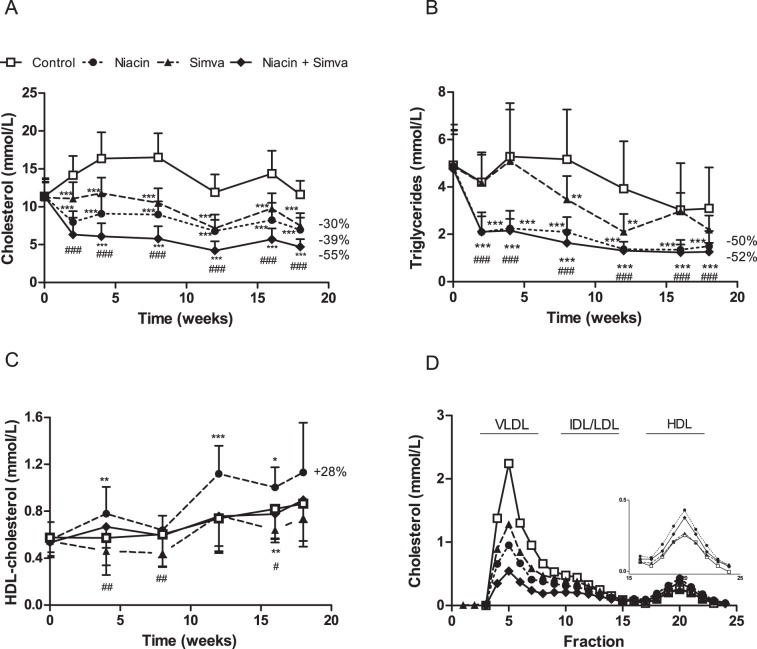
Effect of niacin, simvastatin and their combination on plasma lipid levels. Plasma total cholesterol (A), triglycerides (B) and HDL-cholesterol levels were measured at various time points throughout the study. The average HDL-cholesterol levels were calculated for all the treatment groups (C). Lipoproteins were separated by FPLC and cholesterol was measured in the fractions after 18 weeks of treatment (D). (Simva, simvastatin; values are means ± SD; n = 15 per group; **P<0.01 and ***P<0.001 as compared to control; ^#^P<0.05 and ^###^P<0.001 as compared to niacin+simvastatin).

To verify the lipid-lowering effect of niacin alone and in combination with simvastatin, we measured plasma TC (Figure 1A), TG (Figure 1B) and HDL-C (Figure 1C) levels during the study. The western-type diet resulted in an average TC of 13.4±1.7 mmol/L, TG of 4.3±1.4 mmol/L and HDL-C of 0.65±0.13 mmol/L (control group). TC and TG levels were reduced by niacin (−39%, P<0.001; −50%, P<0.001), simvastatin (−30%, P<0.001; −19%, NS) and the combination (−55%, P<0.001; −52%, P<0.001). The combination reduced TC to a greater extent than simvastatin alone (−36%, P<0.001). Niacin increased HDL-C by +28% (P<0.01) as compared to the control, whereas the combination increased HDL-C by +14% (P<0.05) as compared to simvastatin mono-treatment. Niacin alone resulted in higher HDL-C than the combination (P<0.001). The reductions in plasma TC induced by niacin, simvastatin and the combination were confined to apoB-containing lipoproteins as measured after lipoprotein separation by FPLC (Figure 1D).

### Niacin Reduces ApoB-containing Lipoprotein Cholesterol by Modestly Increasing VLDL Clearance Without Affecting VLDL Production

**Figure 2:**
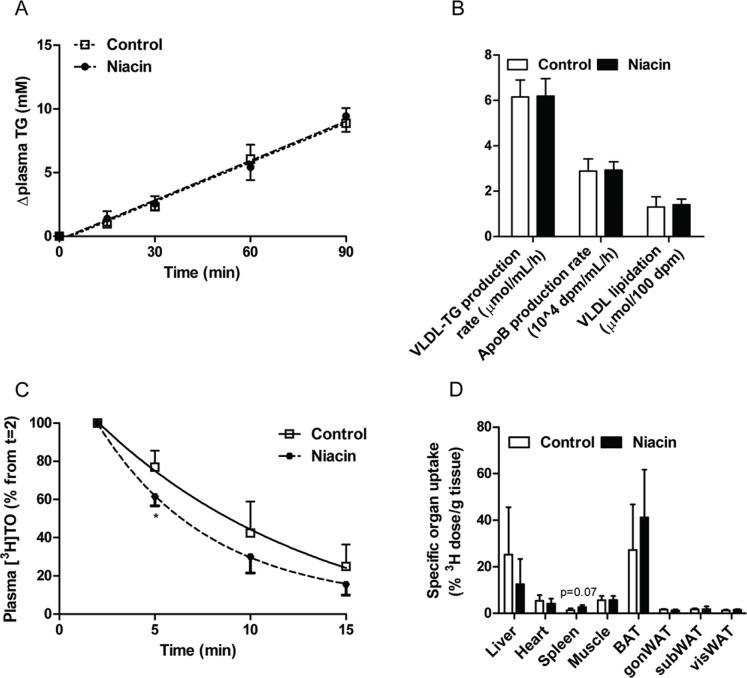
Effect of niacin on VLDL production and clearance. To determine VLDL production, mice were injected with Trans^35^S label and tyloxapol and the accumulation of TG in plasma (A) and the production rate of VLDL-TG and apoB, as well as VLDL lipidation, defined as the ratio of VLDL-TG/apoB, were determined (B). To determine VLDL clearance, mice were injected with glycerol tri[^3^H]oleate- and [^14^C]cholesteryl oleate-labeled VLDL-like emulsion particles. Plasma ^3^H-activity was determined as percentage of the initial dose (C), and uptake of ^3^H-activity by various organs was determined as percentage of the injected dose per gram wet tissue (D). (BAT, brown adipose tissue; gonWAT, gonadal white adipose tissue; subWAT, subcutaneous white adipose tissue; visWAT, visceral white adipose tissue; values are means ± SD; n = 6 per group for VLDL production and n = 3–5 per group for VLDL clearance; *P<0.05 as compared to control).

To determine by which mechanism the level of apoB-containing lipoprotein cholesterol is decreased, first the VLDL-TG production was assessed after injection of Tran^35^S label and tyloxapol. VLDL-TG production did not differ between controls and niacin-treated mice (Figure 2A; control 6.1±0.7 µmol/mL/h versus niacin 6.2±0.8 µmol/mL/h; P = 0.94). In addition, the apoB production rate, as measured by incorporation of ^35^S-activity in the VLDL fraction (Figure 2B; control 2.9±0.5 µmol/mL/h versus niacin 2.9±0.4 µmol/mL/h; P = 0.94) and VLDL-apoB lipidation (control 1.3±0.4 nmol/100 dpm versus niacin 1.4±0.3 nmol/100 dpm; P = 0.69) did not differ between groups.

We then examined the clearance and uptake of [^3^H]TO and [^14^C]CO-labeled VLDL-like emulsion particles. Despite lack of statistical power, due to unexpected loss of mice, there was a trend towards a faster plasma clearance rate of [^3^H]TO (Figure 2C control t_1/2_ = 6.4±2.2 min versus niacin t_1/2_ = 4.9±0.9 min; P = 0.19) and a significantly faster initial [^3^H]TO clearance in the first 5 min after niacin treatment (P<0.05). Tissue-specific ^3^H-accumulation did not differ between groups, although there was a tendency (P = 0.07) for a higher ^3^H-accumulation in the spleen from the niacin-treated mice (Figure 2D). This was accompanied by a non-significant increase in the plasma clearance rate of [^14^C]CO (control t_1/2_ = 11.6±5.5 min versus niacin t_1/2_ = 7.1±1.7 min; P = 0.12) with no differences in ^14^C-accumulation in the various organs between groups (data not shown).

### Niacin, Simvastatin and their Combination Reduce Plasma CETP Activity, CETP Mass and Niacin alone and together with Simvastatin Reduces Hepatic CETP Gene Expression

In a previous study, we showed that niacin increased HDL-C by decreasing hepatic CETP expression and plasma CETP concentration [18]. To verify this, we measured plasma CETP activity and mass and hepatic CETP mRNA expression after 4 and/or 18 weeks of treatment (Table 1). Niacin, which most prominently increased HDL-C, reduced the average plasma CETP activity by −21% (P<0.01) and mass by −22% (P<0.01). Simvastatin reduced CETP activity and mass by −25% and −37%, respectively (both P<0.001) without affecting HDL-C levels. The combination reduced CETP activity (−34%; P<0.001) and mass (−48%; P<0.001) to an even higher extent. Previously, we demonstrated that this reduction was due to reduced CETP mRNA expression in the liver. In line with these results, we found that niacin alone and in combination with simvastatin tended to reduce hepatic CETP expression to −76% (P = 0.072) and −58% (P = 0.059), respectively. Besides the liver, WAT is considered as a major source of CETP and since WAT is a target of niacin [29], we also determined the effect of all treatments on CETP mRNA expression in WAT. Niacin did not decrease CETP expression in WAT (data not shown), which is consistent with a recent study in CETP transgenic mice [30]. For the control group, a 125 times lower relative CETP expression was measured in WAT compared to the liver (data not shown). A mild correlation was found between hepatic CETP expression and plasma CETP mass (R^2^ = 0.25, P = 0.006), whereas CETP expression in WAT did not correlate with plasma CETP mass (R^2^ = 0.02, P = 0.45) (data not shown). Taken together, we concluded that the liver is the major determinant for circulating CETP levels in this model, which were affected by all the treatments.

**Table 1:** The effect of niacin, simvastatin and their combination on plasma CETP activity after 4 and 18 weeks of treatment, as well as plasma CETP mass and hepatic CETP expression after 18 weeks of treatment.

	Average plasma CETP activity(nmol/mL/h)	Plasma CETP mass (µg/mL)	Hepatic CETP expression(% of control)
Control	64.3±11.4	21.3±3.4	100±30
Niacin	50.6±6.6 **	16.6±3.5 **	76±23 P = 0.072
Simva	48.1±8.4 ***	13.4±3.5 ***	76±25
Niacin+Simva	42.6±8.9 *** P = 0.081	11.0±2.2 *** P = 0.050	58±33 P = 0.059

CETP, cholesteryl ester transfer protein; Simva, simvastatin. Values are means ± SD (n = 15 per group for plasma CETP activity and mass and n = 6–8 per group for hepatic CETP expression). **P<0.01 and ***P<0.001 as compared to control.

### Niacin alone and in Combination with Simvastatin Reduces Atherosclerosis Development to a Greater Extent than Simvastatin Treatment alone

**Figure 3:**
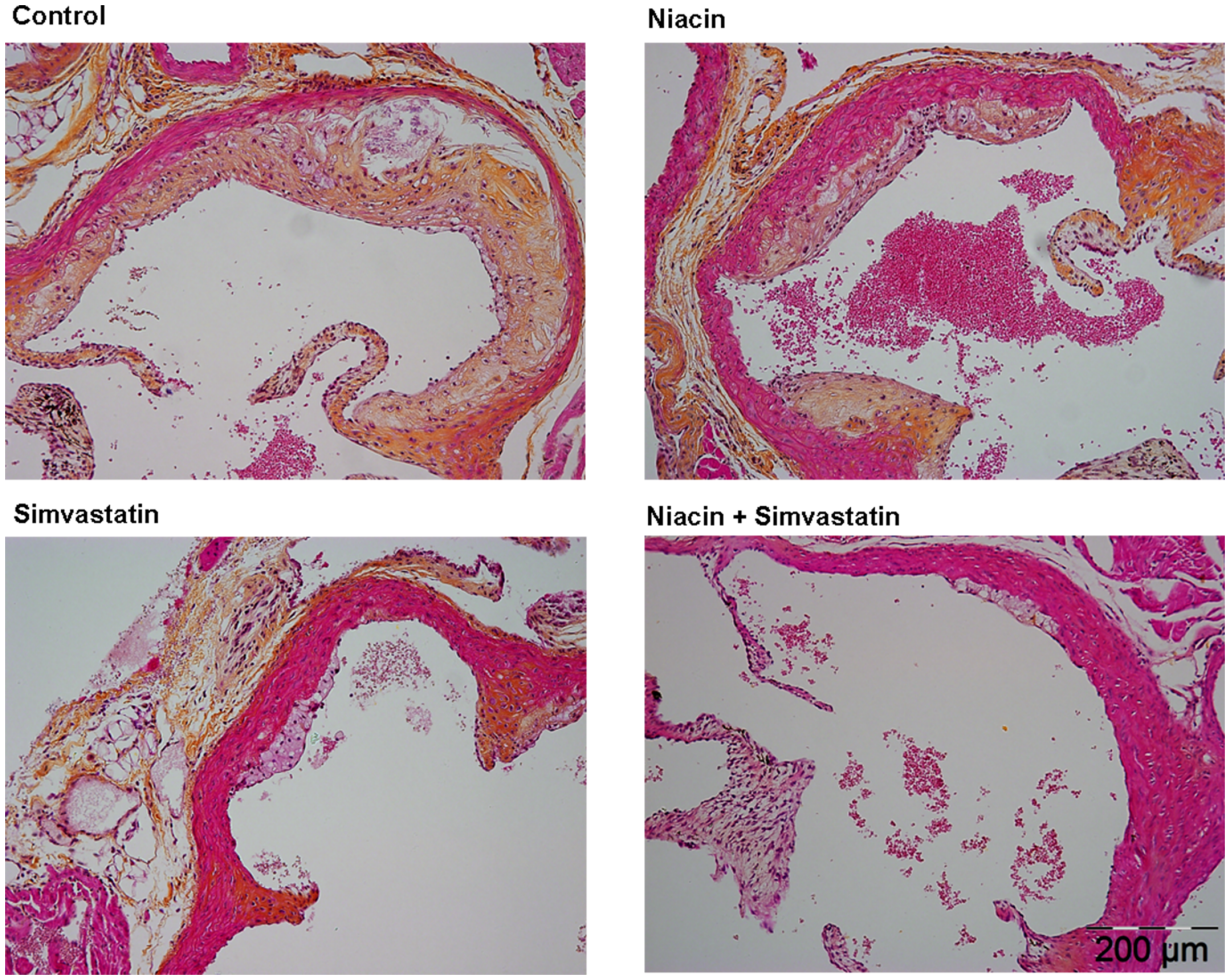
Effect of niacin, simvastatin and their combination on plaque morphology. Representative images of hematoxylin-phloxine-saffron-stained atherosclerotic lesions in a cross section of the aortic root area for the control group (A), niacin group (B), simvastatin group (C) and the combination group (D) after 18 weeks of treatment.

**Figure 4:**
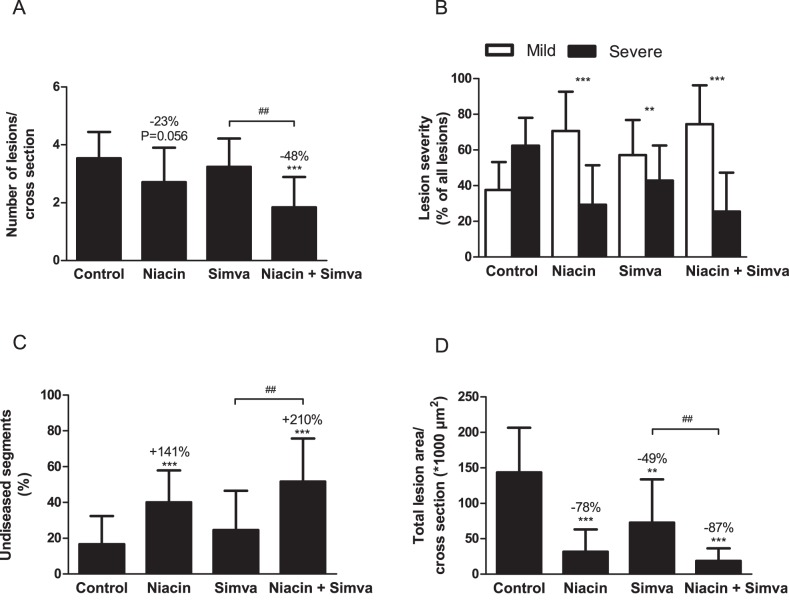
Effect of niacin, simvastatin and their combination on atherosclerosis development in aortic root area. After 18 weeks of treatment, number of lesions (A), lesion severity (B), percentage undiseased segments (C) and total lesion area (D) were determined per cross section. Lesion severity was classified as mild (type I–III) and severe (type IV–V) lesions. (Simva, simvastatin; values are means ± SD; n = 15 per group; **P<0.01 and ***P<0.001 as compared to control; ^##^P<0.01 as compared to niacin+simvastatin).

After 18 weeks of treatment, we measured the effect of niacin, with and without simvastatin on atherosclerosis development in the aortic root. Figure 3 illustrates representative images of atherosclerotic lesions for each group. We determined the number of lesions per cross section (Figure 4A), the lesion severity as a percentage of all lesions (Figure 4B), the percentage undiseased segments (Figure 4C) and the total lesion area per cross section (Figure 4D). To determine lesion severity as a percentage of all lesions, type I-III lesions were classified as mild lesions and type IV–V lesions were classified as severe lesions.

In the control group, a fair amount of atherosclerosis developed with 3.5±0.9 lesions per cross section, of which 62±16% were severe and only 17±16% undiseased segments. The total lesion area was 144±63×10^3^ µm^2^ per cross section. When compared to the control, niacin reduced the number of lesions (−23%; P = 0.056), attenuated lesion severity (P<0.001) and increased the percentage undiseased segments (+141%, P<0.001). Furthermore, niacin strongly decreased the total lesion area by −78% (P<0.001). Simvastatin alone was less effective as it only reduced lesion severity (P<0.01) and total lesion area (−49%, P<0.01). The combination had potent inhibiting effects on lesion development, as evidenced by reductions in lesion number (−48%, P<0.001), severity (P<0.001) and area (−87%, P<0.001), and by an increase in the percentage of undiseased segments (+210%, P<0.001). Furthermore, the percentage undiseased segments, as well as the reduction in the lesion number and area was greater after the combination compared to simvastatin alone (−44%; +110%; −74%, all P<0.01). These results showed that niacin mono-treatment was very potent in inhibiting atherosclerotic lesion development in APOE*3Leiden.CETP mice and that niacin added to the atherosclerosis-reducing effects of simvastatin.

### Niacin Improves Lesion Stability Index and Decreases Functional Markers of Vascular Inflammation

**Figure 5:**
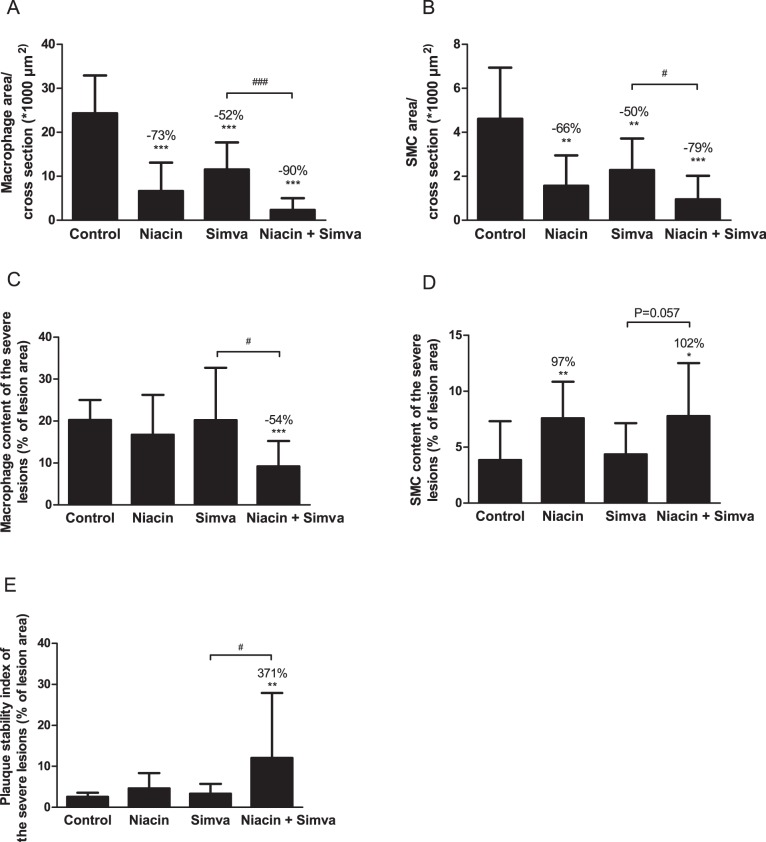
Effect of niacin, simvastatin and their combination on lesion composition. Macrophage area (A) and SMC area (B) were determined for all lesions and calculated per cross section. To correct for lesion size, macrophage content (C), SMC content (D), as well as plaque stability index (ratio of collagen and SMC content to macrophage content) (E) were also calculated as a percentage of lesion area, specifically in severe lesions (Type IV–V). (Simva, simvastatin; SMC, smooth muscle cells; values are means ± SD; n = 15 per group; *P<0.05, **P<0.01 and ***P<0.001 as compared to control; ^#^P<0.05, and ^###^P<0.001 as compared to niacin+simvastatin).

After investigation of lesion morphology, we analyzed the treatment effects on plaque composition. For all lesions, the macrophage area as destabilization factor (Figure 5A), as well as SMC (Figure 5B) and collagen area (data not shown) as stabilization factors were calculated per cross section. The macrophage, SMC and collagen area per cross section in the control group were 24.3±8.6×10^3^ µm^2^, 4.6±2.3×10^3^ µm^2^ and 64.2±37.0×10^3^ µm^2^, respectively. All treatments reduced the macrophage (−73%, −52% and −90%; all P<0.001) and the SMC area (−66%, P<0.01; −50%, P<0.01; −79%, P<0.001). As a measure of the lesion stability index, the ratio of collagen and SMC area (i.e. stabilization factors) to macrophage area (i.e. destabilization factor) was determined for all lesions (data not shown). The lesion stability ratio for the control group was 2.7±1.4. Combination treatment tended to increase this ratio by +201% (P = 0.085).

After finding indications for more stable lesions, we specifically focused on the composition of the more severe lesions, which are considered to be the most vulnerable lesions. Additionally, we corrected for the lesion area. Thus, we measured the macrophage (Figure 5C), SMC (Figure 5D) and collagen content (data not shown) as a percentage of lesion area. The severe lesions in the control group consisted of 20% macrophages, 4% SMC and 45% collagen (latter data not shown). Niacin alone and in combination with simvastatin reduced the relative macrophage content by -18% (NS) and −54% (P<0.001), respectively and increased the relative SMC content by +97% (P<0.01) and +102% (P<0.05), respectively. The combination was superior to simvastatin mono-treatment in stabilizing the plaque as seen by a reduction in macrophage content (−54%; P<0.05) and an increase in SMC content (+79%; P = 0.057). There were no significant differences in collagen content between groups (data not shown). The lesion stability index was also calculated for the severe lesions based on the relative area (Figure 5E). Combination treatment increased this ratio by +371% (P<0.01) compared to the control (2.6±1.0) and to a greater extent compared to simvastatin (P<0.05) alone.

**Figure 6:**
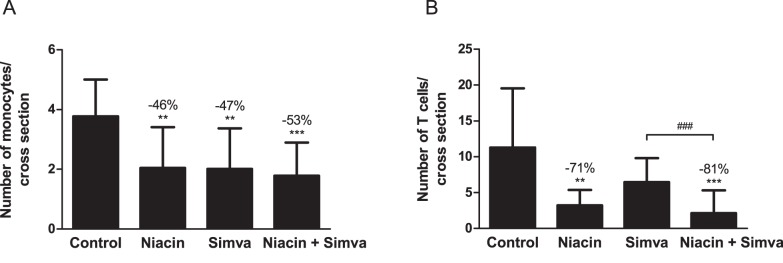
Effect of niacin, simvastatin and their combination on monocyte adhesion and T cell number. The number of monocytes adhering to the endothelium (A) and the number of T cells in the aortic root area (B) were determined per cross section. (Simva, simvastatin; values are means ± SD; n = 15 per group; **P<0.01; ***P<0.001 as compared to control; ^###^P<0.001 as compared to niacin+simvastatin).

As functional markers of vessel wall inflammation, the number of monocytes adhering to the activated endothelium (Figure 6A) and T cells in the aortic root area (Figure 6B) were counted and calculated per cross section. In the control group, 3.8±1.2 adhering monocytes and 11.3±8.3 T cells were present. A marked reduction of adhering monocytes of more than −45% (P<0.01; P<0.01 and P<0.001, respectively) was found in all treatment groups, where T cells abundance was reduced by niacin alone (−71%, P<0.01) and in combination with simvastatin (−81%, P<0.001).

### Niacin Reduces Atherosclerosis Progression Primarily by Reducing NonHDL-Cholesterol

**Figure 7:**
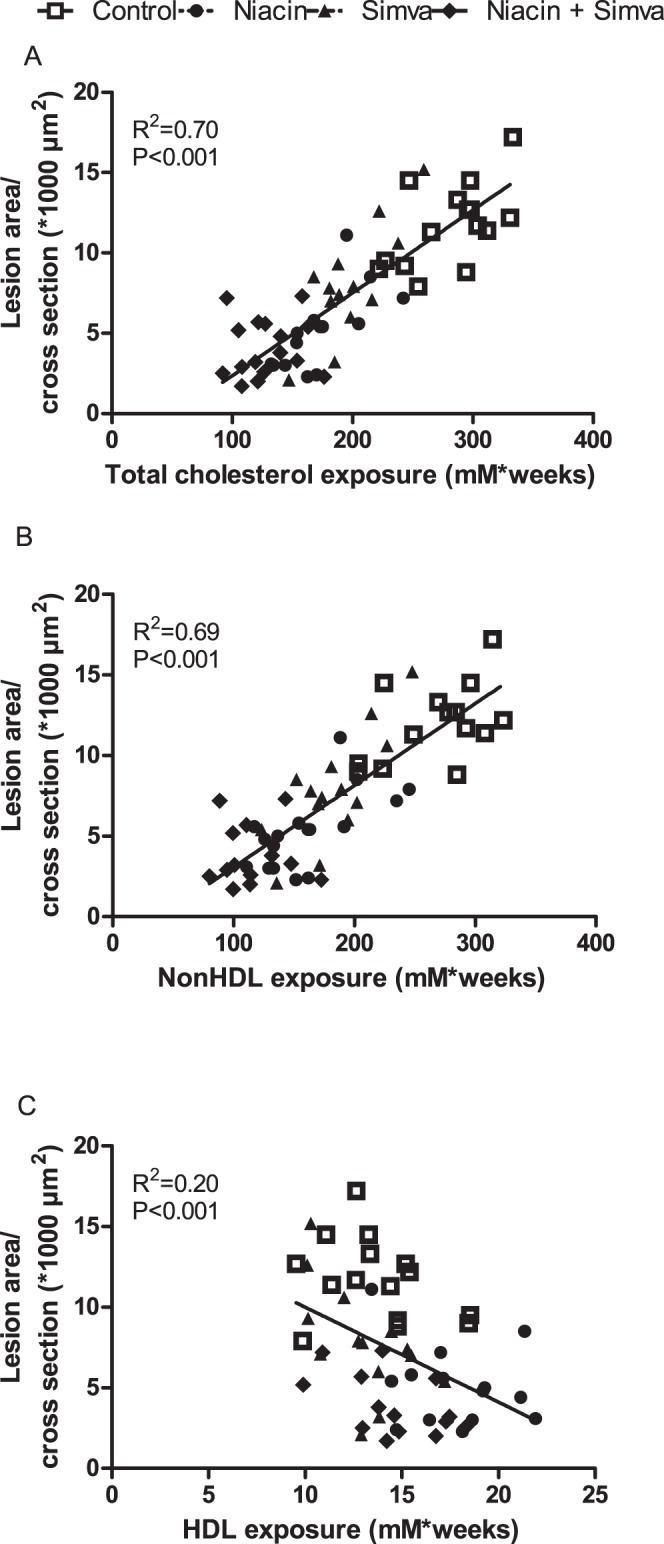
Correlation between plasma cholesterol exposure and lesion area. The square root of the lesion area was plotted against total cholesterol exposure (A), nonHDL-cholesterol exposure (B) and HDL-cholesterol exposure (C). Linear regression analyses were performed. (Simva, simvastatin; n = 15 per group).

In addition to inflammation, plasma cholesterol is certainly a strong determinant for atherosclerosis progression. We evaluated whether the anti-atherogenic effect of niacin and simvastatin could be explained by the reduction in plasma TC (Figure 7). Since atherosclerotic lesion area showed a quadratic dependence on plasma TC exposure, lesion area was transformed using a square root transformation. Lesion area was strongly predicted by plasma TC exposure (R^2^ = 0.70, P<0.001; Figure 7A), and nonHDL-C exposure (R^2^ = 0.69, P<0.001; Figure 7B) and to a much lesser extent by HDL-C exposure (R^2^ = 0.20, P<0.001; Figure 7C). Together, nonHDL-C and HDL-C exposure accounted for 71% of the variability in lesion area and predicted the lesion area independently of each other (P<0.001 and P<0.05, respectively). Importantly, the effects of niacin and simvastatin on lesion area were lost after correcting for both HDL-C and nonHDL-C exposure (P = 0.16; P = 0.61, respectively). Furthermore, no effect of niacin and simvastatin on monocyte adhesion was found after correcting for nonHDL-C exposure (P = 0.50; P = 0.20, respectively). However, niacin decreased the square-root transformed macrophage area and T cell abundance even after correcting for nonHDL-C exposure (both P<0.01), whereas simvastatin did not (P = 0.12; P = 0.26, respectively).

Collectively, these data are compatible with a mechanism that niacin and simvastatin mainly decrease atherosclerotic lesion development via a reduction of nonHDL-C with an additional effect of HDL-C-elevation for niacin, while a direct effect on lesion macrophages and T cell abundance may contribute to the anti-atherogenic effect of niacin, but not simvastatin.

### Niacin Mildly Increases Reverse Cholesterol Transport

**Figure 8:**
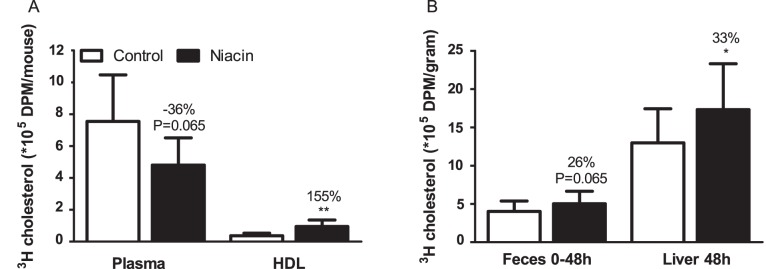
Effect of niacin on reverse cholesterol transport. [^3^H]-cholesterol-labeled macrophages were injected in control and niacin-treated mice and ^3^H activity was determined in plasma and HDL (A) and the liver 48 h after injection, as well as in feces collection between 0–48 h after injection (B). (Values are means ± SD; n = 8 per group; *P<0.05 and ** P<0.01 as compared to control).

To investigate the possible mechanism by which the HDL-C-raising effect of niacin may contribute to its anti-atherogenic effect, we performed an *in vivo* RCT experiment. After 3 weeks of treatment, mice were injected with [^3^H]-cholesterol-labeled macrophages. Forty eight hours after injection, plasma total ^3^H-activity tended to be decreased (−36%, P = 0.065), whereas ^3^H-activity in the HDL fraction was increased after niacin treatment (+155%, P<0.01; Figure 8A). In addition, niacin increased ^3^H-activity in the liver (+33%; P<0.05) and tended to increase fecal ^3^H-activity (+26%; P = 0.065; Figure 8B).

## Discussion

In this study, we aimed to address the discrepancy between the beneficial effects of niacin in initial clinical trials [3]–[6], [14] and the lack of effect of niacin on top of statin treatment on reduction of cardiovascular events in the AIM-HIGH [8] and HPS2-THRIVE [7] trials by investigating the effects of niacin without and with simvastatin on atherosclerosis development and determine the underlying mechanisms in APOE*3Leiden.CETP mice, a mouse model for familial dysbetalipoproteinemia (FD). We demonstrated that niacin decreased atherosclerosis development mainly by reducing nonHDL-C with a modest HDL-C-raising and anti-inflammatory effect and that the additive effect of niacin on top of simvastatin was mostly dependent on its nonHDL-C-lowering capacities.

First, we showed that niacin and simvastatin both reduced plasma lipid levels. Niacin reduced (V)LDL-C and (V)LDL-TG and increased HDL-C, whereas simvastatin mainly reduced (V)LDL-C. Combination treatment of niacin and simvastatin reduced nonHDL-C more effectively as compared to simvastatin alone. In our study, the reduction in plasma TC after niacin treatment alone and in combination with simvastatin was greater compared to recent clinical trials. The APOE*3Leiden mouse was initially developed as an animal model for FD or type III hyperlipoproteinemia, which is characterized by elevated levels of cholesterol and an increased ratio of cholesterol to TG in the VLDL and intermediate density lipoprotein (IDL) fractions, resulting in the appearance of β-VLDL particles [31], [32]. Similarly as in FD patients, in APOE*3Leiden and APOE*3Leiden.CETP mice as a model for mixed dyslipoproteinemia, a major part of plasma cholesterol is contained in the VLDL and VLDL-remnant particles, leading to formation of β-VLDL particles, which further increases after cholesterol feeding. Whereas niacin reduces plasma TC by ∼5–15%, LDL-C by ∼5–20% and TG by ∼15–30% in patients with hyperlipidemia [2], in two small studies in FD patients, niacin decreased TC by 23–50% and TG by 43–62% [33], [34] with -56% reduction of VLDL-C and 48% reduction of VLDL-TG [34]. Thus, the extent of lipid-lowering observed with niacin in APOE*3Leiden.CETP mice is comparable to that of FD patients.

Since plasma VLDL-TG and apoB levels are determined by the balance between VLDL production and clearance, we evaluated their individual contribution. VLDL production was not affected in niacin-treated mice, neither was apoB production or lipidation of the VLDL particle. However, a modest effect of niacin on VLDL clearance was observed. The mechanism behind the lipid-lowering effect of niacin has been generally ascribed to a reduced hepatic VLDL production, as a result of decreased free fatty acid (FFA) flux from WAT after inhibition of hormone sensitive lipase. However, an initially decreased FFA flux is followed by a rebound effect with increased release of FFA [1], [35]. In humans, contradicting data describe that niacin decreased VLDL production without affecting VLDL clearance [36]–[38] and on the other hand that niacin enhanced clearance of apoB without affecting production [39]. The latter study is in line with our results, which implicate VLDL clearance rather than VLDL production as the possible mechanism by which niacin reduces apoB.

The niacin-induced increase in HDL-C in the present study may be attributed to a decrease in hepatic and plasma CETP leading to an inhibition of HDL delipidation as previously described [18]. As similar effects on CETP levels and activity were observed after simvastatin treatment, without affecting HDL, different mechanisms are likely involved in either treatment. Interestingly, a decreased macrophage content accompanying decreased hepatic cholesterol accumulation as a result of niacin’s lipid-lowering effect was recently proposed as a mechanism by which niacin decreases hepatic CETP expression [30].

An important observation from our studies is that niacin decreases atherosclerosis progression and adds to the anti-atherogenic effect of simvastatin, in particular regarding its enhancing effect on plaque stability. Niacin decreased lesion number, severity and area, and increased the percentage undiseased segments. Moreover, niacin improved lesion composition by reducing the macrophage content and increasing the SMC content. Importantly, the combination treatment increased the plaque stability index, defined as the ratio of SMC and collagen over macrophage area, as compared to either niacin or simvastatin alone.

It is interesting to speculate on the mechanism(s) underlying the anti-atherogenic effect of niacin. In the APOE*3Leiden.CETP mouse model, statistical analyses revealed that the effects of niacin and simvastatin were largely explained by their reduction in nonHDL-C, as evidenced by a strong correlation between plasma nonHDL-C and lesion area. The fact that the combination treatment reduced nonHDL-C beyond the level reached by simvastatin alone can thus largely explain why niacin added to the anti-atherosclerotic effect of simvastatin. Though, HDL-C also appeared to predict lesion area independent of nonHDL-C, albeit that the predictive value of HDL-C was much smaller than that of nonHDL-C. The HDL-C-raising effect of niacin, may, therefore, have contributed to some extent to the anti-atherosclerotic activity of niacin.

To explore the contribution of the niacin-induced increase in HDL-C to the reduction of atherosclerosis, we investigated the functionality of HDL by performing an RCT experiment. From this experiment, we conclude that the effect of niacin on RCT may partially contribute to, but is not the driving force behind its anti-atherogenic effects. This is in accordance with our statistical correlations, which showed nonHDL-C to be a much stronger contributor to atherogenesis. Although HDL-C contributed to some extent, we observed that niacin’s attenuating effect on atherosclerosis development in APOE*3Leiden.CETP mice, fed a Western-type diet with 0.1% cholesterol, is largely explained by its lipid-lowering effect. At first sight, this seems to contradict a recent report showing that niacin reduced atherosclerosis development in LDL receptor-deficient mice under conditions that left plasma cholesterol levels unaffected [15]. In that mouse model, the atheroprotective effects of niacin were mainly explained by impaired homing macrophage recruitment to atherosclerotic plaques and by promoting cholesterol efflux from macrophages by upregulation of ABCG1. However, it should be noted that those mice were fed a high fat diet containing as much as 1.5% cholesterol. A previous study from our laboratory showed that dietary cholesterol induced dose-dependent marked inflammation in mice [40], where the liver switches to an inflammatory, pro-atherosclerotic state as reflected by a strong increase in serum amyloid A levels at dietary cholesterol levels exceeding 0.5%. Previously, Lukasova *et al*. [15] evaluated the anti-atherogenic effect of niacin under highly inflammatory conditions, at which the anti-inflammatory properties of niacin may become dominant and may not necessarily reflect the mode of action for niacin under mild cholesterol intake as used in the present study.

It should be noted that we also obtained evidence that niacin exerted anti-inflammatory effects in our mouse model under milder dietary conditions. Firstly, niacin reduced monocyte adhesion and macrophage area of the atherosclerotic lesions. In fact, niacin reduced macrophage area independent of nonHDL-C, whereas simvastatin did not. These data not only corroborate the findings in LDL receptor-deficient mice [15], but also the recent observations that niacin reduced collar-inflicted vascular inflammation and inhibited intima-media neutrophil recruitment in New Zealand White rabbits independent of changes in plasma lipids [41]. Secondly, we observed that niacin, but not simvastatin, strongly reduced the number of T cells in the aortic root area, which are involved in the progression of atherosclerosis [42]. The reduction was independent of nonHDL-C exposure, suggesting the anti-inflammatory effect observed was brought about by niacin, instead of HDL derived. This is in accordance with a study where niacin inhibited monocyte chemotactic protein 1 (MCP-1), RANTES and fractalkine in adipocytes. These chemokines contribute to the recruitment of T cells and macrophages. WAT is known to express the GPR109A receptor and has the ability to contribute to both systemic and local (perivascular) inflammation associated with atherosclerosis [29], [43].

Although initial clinical studies showed that niacin reduced atherosclerosis development in combination with statins [3]–[5] and reduced the relative risk of cardiovascular events [6], results from the large outcome trials AIM-HIGH and HPS2-THRIVE did not confirm earlier findings [8], [44], [45]. In order to test the HDL hypothesis, the AIM-HIGH investigators minimized the differences in LDL levels between the groups. Patients enrolled in the trial were subjected to aggressive LDL-C-lowering treatment, aimed at LDL-C of 40–80 mg/dL (1.03–2.07 mmol/L), reaching mean baseline LDL-C of 71 mg/dL (1.84 mmol/L) and HDL-C of 35 mg/dl (0.91 mmol/L) [46]. A modest increase in HDL-C was observed in the placebo group, resulting in a 4–5 mg/dl (0.10–0.13 mmol/L) difference in HDL-C between groups. This, together with the aggressive LDL-C-lowering may have given rise to insufficient power to detect a reduction in events [45]. Unexpectedly, the much larger outcome trial HPS2-THRIVE failed to reveal further cardiovascular risk reduction when adding ER-niacin/laropiprant to vigorous statin treatment, plus if required ezetimibe, as compared to statin/(ezetimibe) mono-treatment [7]. Furthermore, there was a significant increase in non-fatal serious adverse events and drop-out rate in the ER-niacin/laropiprant-treated patients. The lack of inclusion criteria for HDL-C resulted in a baseline HDL-C of >40 mg/dl (1.14 mmol/L) with a low baseline LDL-C level of <70 mg/dl (1.64 mmol/L). Patient stratification revealed that baseline HDL-C levels did not predict efficacy of niacin and that indeed only in patients with high LDL-C niacin reduced major cardiovascular events.

In conclusion, our results show that niacin decreases atherosclerosis development mainly by reducing nonHDL-C with modest HDL-C-raising and additional anti-inflammatory effects. The additive effect of niacin on top of simvastatin is mostly dependent on its nonHDL-C-lowering capacities. These data suggest that clinical beneficial effects of niacin are largely dependent on its ability to lower LDL-C on top of concomitant lipid-lowering therapy and may explain the failure of niacin in the clinical outcome trials.

## Supporting Information

Table S1 **RT-PCR primer sequences.**
(DOCX)
