# Supplementary material for: Niacin Reduces Atherosclerosis Development in APOE*3Leiden.CETP Mice Mainly by Reducing NonHDL-Cholesterol
Source: PLoS One. 2013 Jun 19;8(6):e66467. doi: 10.1371/journal.pone.0066467 (PMC3686722; doi:10.1371/journal.pone.0066467)
Supplement: Table S1 — RT-PCR primer sequences. (DOCX) [file pone.0066467.s001.docx]

**Supporting Information**

Table S1. RT-PCR primer sequences

| Gene | Forward primer | Reverse primer |
| --- | --- | --- |
| HPRT | TTGCTCGAGATGTCATGAAGGA | AGCAGGTCAGCAAAGAACTTATAG |
| 36B4 | GGACCCGAGAAGACCTCCTT | GCACATCACTCAGAATTTCAATGG |
| CETP | CAGATCAGCCACTTGTCCAT | CAGCTGTGTGTTGATCTGGA |

HPRT, hypoxanthine-guanine phosporibosyltransferase; 36B4, acidic ribosomal phosphoprotein PO; CETP, cholesteryl ester transfer protein.
